# H-NS Silences Gene Expression of LeuO, the Master Regulator of the Cyclic(Phe-Pro)-dependent Signal Pathway, in *Vibrio vulnificus*

**DOI:** 10.4014/jmb.2001.01021

**Published:** 2020-03-27

**Authors:** Na-Young Park, Keun-Woo Lee, Kun-Soo Kim

**Affiliations:** Department of Life Science, Sogang University, Seoul 04107, Republic of Korea

**Keywords:** *Vibrio vulnficus*, H-NS, cyclic(Phe-Pro), ToxR, LeuO, gene regulation

## Abstract

The histone-like nucleoid structuring protein (H-NS) is an abundant global regulator of environmentally controlled gene expression. Herein, we demonstrate that H-NS represses the expression of LeuO, the master regulator of the cyclic(Phe-Pro)-dependent signaling pathway, by directly binding to the upstream region of the gene. H-NS binds to a long stretched region (more than 160-bp long), which overlaps with binding sites for ToxR and LeuO. A high quantity of H-NS outcompetes ToxR for binding to the *cis*-acting element of *leuO*. However, our footprinting analyses suggests that the binding of H-NS is relatively weaker than LeuO or ToxR at the same molarity. Considering that the DNA nucleotide sequences of the upstream regions of *leuO* genes are highly conserved among various *Vibrio*, such patterns as those found in *V. vulnificus* would be a common feature in the regulation of *leuO* gene expression in *Vibrio*naceae. Taken together, these results suggest that, in species belonging to *Vibrio*naceae, H-NS regulates the expression of *leuO* as a basal stopper when cFP-ToxR mediated signaling is absent.

## Introduction

The histone-like nucleoid structuring protein (H-NS) is a small highly abundant protein that functions as a nucleoid organizer and compacts DNA [1]. H-NS acts as a global regulator of genes generally controlled by environmental signals [2]. H-NS belongs to the nucleoid-associated protein family, which includes the factor for inversion stimulation (Fis), integration host factor (IHF), the heat-stable protein HU, and the leucine-responsive protein (Lrp) [3]. It functions as a transcriptional silencer at promoters exhibiting highly curved AT-rich DNA [3]. In *V. cholerae*, H-NS has been reported to down-regulate virulence gene expression at multiple levels in the ToxR regulatory cascade by repressing transcription [4].

*Vibrio* species such as *V. vulnificus* and *V. cholerae* produce diffusible signal molecule cyclo(Phe-Pro) (cFP) [5, 6]. cFP functions as a signal to regulate genes associated with the virulence of the pathogen. The cFP-dependent signaling pathway consists of a series of components. cFP triggers signaling by physically binding to the periplasmic domain of ToxR. The binding consequently causes a change in the cytoplasmic domain of the protein, and induces the expression of *ompU* and *leuO* [7]. However, LeuO, at high levels, self-represses the expression of its own coding gene by binding to the upstream region of the gene, maintaining a certain level of expression via feedback inhibition [7]. LeuO is the master regulator for inducing the expression of vHU*α* and *β*, the bacterial histone-like proteins. These factors, in turn, stabilize the mRNA of the alternative sigma factor RpoS, which enhances the expression of virulence factors, including KatG, a catalase [8]. This enzyme increases survival of *V. vulnificus* under oxidative stress exerted by reactive oxygen species generated by animal host cells, which was induced by cFP produced by infecting *V. vulnificus* [8, 9]. In this signaling pathway, the expression level of the master regulator LeuO is of crucial importance for affecting the expression of numerous virulence factors controlled by each regulatory component; LeuO itself, vHU*αβ*, and RpoS.

We reported that expression of LeuO is controlled by numerous *cis*- and *trans*-elements, forming a complicated regulatory network [7]. LeuO is an LysR-type transcriptional regulator (LTTR), which comprises a large group of transcriptional regulators in prokaryotes [10, 11]. They are highly conserved and have been identified in many bacteria such as *Escherichia coli*, *Salmonella enterica serovar Typhimurium*, *Rhizobium* spp., *Enterobacter cloacae*, and *Vibrio* spp [12]. The binding sequence for LTTR, generally called LTTR box, is T-N_11_-A, which has high A+T rich sequences [12, 13]. These high A+T rich sequences are known to be related to DNA bending [12]. Moreover, such sequences are known to be bound by H-NS [14]. These led us to hypothesize that the H-NS may also be involved in the regulation of the *leuO* expression in *V. vulnificus*, and here we report that H-NS regulates the expression of *leuO* by direct binding to regions overlapping with those for the binding of other *trans*-acting elements in the upstream region in this human pathogen.

## Materials and Methods

### Strains, Culture Conditions, and Chemicals 

The strains and plasmids used in this study are listed in Supplementary Table S1. *E. coli* strains were cultured in LB medium with appropriate antibiotics at 37°C. *V. vulnificus* strains were cultured in LB medium or in thiosulfate citrate bile salt sucrose (TCBS) agar (Difco, Detroit, MI) at 30°C. All media were purchased from Difco (Detroit, MI). Cyclo phenylalanine proline (cFP) (Bachem Inc, Switzerland) was dissolved in dimethyl sulfoxide (DMSO) and used at a final concentration of 5 mM.

### Construction of *leuO* Deletion in *V. vulnificus* MO6-24/O

To construct a *leuO* deletion mutant, DNA fragments comprising the upstream and downstream regions of *leuO* were amplified using the primers d*leuO*-up-F and d*leuO* -up-R, and the primers d*leuO*-down-F and d*leuO*-down-R, respectively. After confirming the sequences, each fragment was cloned to an SacI-digested pDM4 plasmid [15] using an In-fusion HD cloning kit (Clonetech Laboratories, TaKaRa Bio, Inc., Shiga, Japan) to generate pDM4-d*leuO*. Then the plasmid was introduced into *E. coli* S17-1 λpir [16] to be mobilized into the *V. vulnificus* strain MO6-24/O by conjugation. Double crossover selection to construct the chromosomal deletion of *leuO* was performed as described previously [15]. The deletion was confirmed by PCR and DNA sequencing.

### Construction of *hns* Deletion, *leuO*/*hns* Double Deletion, and *toxRS*/*hns* Triple Deletion in *V. vulnificus* MO6-24/O

To construct a *hns* deletion mutant, DNA fragments comprising the upstream and downstream regions of *hns* were amplified using primers d*hns*-up-F and d*hns*-up-R, and primers d*hns*-down-F and d*hns*-down-R, respectively. The fragments were ligated to the pGEM-T Easy vector (Promega, Madison, WI) to generate pGEM-hns-up and pGEM-hns-down, respectively. pGEM-hns-up was digested with sphI and BamHI, and pGEM-hns-down was digested with BamHI and XhoI. The resulting DNA fragments containing the *hns* upstream and downstream regions were ligated to the pGEM-T Easy vector to construct pGEM-d*hns*. The resulting construct had a 348-bp deletion in *hns*. The plasmid pGEM-d*hns* was digested with sphI and XhoI and ligated to pDM4 [15] to obtain pDM4-d*hns*, which was then introduced into *E. coli* S17-1 λpir [16] to be mobilized into *V. vulnificus* strains MO6-24/O, MO6-d*leuO*, and MO6-d*tox*RS by conjugation. Double crossover selection to construct the chromosomal deletion of *hns* was performed as described previously [15]. The deletion was confirmed by PCR and DNA sequencing, and the mutants were named MO6-d*hns*, MO6-d*leuO*/*hns*, and MO6-d*tox*RS/*hns*, respectively.

### β-Galactosidase Assays

β–Galactosidase activity was measured by methods previously described [17]. Briefly, *V. vulnificus* strains were cultured overnight in LB medium and then washed twice with fresh LB medium. Then cells were subcultured in fresh LB medium. To assess the effect of cFP, either 5 mM cFP, or DMSO as a negative control, was added and samples were diluted to an A_600_ of 0.005. To assess the effect of LeuO by L-arabinose induction, the culture was split into four aliquots after reaching an A_600_ of 0.4, and then various concentrations of L-arabinose were added (0, 0.001, 0.01, 0.1%). The β–Galactosidase activity was measured.

### Construction of an L-Arabinose-Inducible H-NS Expression System

The 408-bp DNA fragment of the *hns* gene was amplified using primers BAD-hns-F and BAD-hns-R. The resulting fragment was cloned into pBAD-TOPO (Invitrogen, Thermo Fisher Scientific Inc., MA) to generate pBAD-hns. A 745-bp DNA fragment, including the *ara* promoter region fused to the upstream of promoter-less *leuO*, was amplified with primers pBAD-F and pBAD-R, and cloned into the EcoRI-digested pBBR1-MCS2 vector [18] using the In-fusion HD cloning kit (Clonetech Laboratories, TaKaRa Bio, Inc., Shiga, Japan) to generate pBBR12-*hns*-ara.

### ChIP Analysis

ChIP analysis was performed in accordance with the manufacturer’s instructions (EZ ChIP, UpstateBiotechnology, Lake Placid, NY) with a minor modification [7]. Briefly, the *toxRS*/*hns* deletion mutants of *V. vulnificus* harboring pRK-toxR-strep-tag and pBBR12-*hns*-ara were grown overnight in LB broth, and sub-cultured to an A_600_ of 0.005 in fresh LB broth. When the growth reached an A_600_ of 0.4, either 0% or 0.1% of L-arabinose was added and the culture was allowed to continue until the growth had reached an A_600_ of 2.0. The experiment was performed as described previously [7]. DNA was analyzed by quantitative real-time PCR (qRT-PCR) on a Light Cycler 480 II real-time PCR system (Roche Applied Science, Upper Bavaria, Germany). qRT-PCR was carried out in triplicate in a 96-well plate (Roche Applied Science) using the primers shown in Supplementary Table S2. Quantification was carried out using the Light Cycler 480 II real-time PCR system software program.

### Expression and Purification of H-NS

DNA fragments encoding 136 amino acids of H-NS was PCR-amplified using the primers pet-hns-F and pet-hns-R. The amplified fragment was cloned into pET21a (Novagen, Madison, WI) using the restriction enzymes BamHI and salI to construct pET-HNS. The resulting construct encoded H-NS fused with a His-tag at the C-terminus. This plasmid was transformed into *E. coli* BL21 (DE3) (Novagen, Madison, WI). The pET-HNS was cultured overnight in LB and then cells were subcultured in fresh LB medium. The expression was induced with 1mM IPTG at A_600_ of 0.4 . After centrifugation, bacterial pellets were suspended in 1× binding buffer (0.5 M NaCl, 5 mM imidazole, 20 mM Tris-HCl, pH 8.0) (Novagen, Madison, WI), sonicated, and then centrifuged at 13,000 rpm for 10 min. The supernatant was applied to His·Bind resin (Novagen, Madison, WI), and bound proteins were eluted with 1× elution buffer (1 M imidazole, 0.5 M NaCl, 20 mM Tris-HCl, pH 8.0). The purity of the eluted protein was confirmed by 15% SDS-PAGE.

### Purification of Cytoplasmic Domain of ToxR and LeuO

The purification of cytoplasmic domain of ToxR (ToxR-N) and LeuO were performed as described previously [7]. Briefly, the pET-ToxR-N was induced with 1 mM IPTG at A_600_ of 0.4 and pRE1-*leuO* was induced by adding tryptophan (final concentration 100 μg/ml) (Sigma) in M9 salt-based induction medium (0.2% casamino acids, 1% glycerol, and M9 salts) at A_600_ of 0.4. After centrifugation, bacterial pellets were suspended in 1 × binding buffer (0.5 M NaCl, 5 mM imidazole, 20 mM Tris-HCl, pH 8.0) (Novagen, Madison, WI), sonicated, and centrifuged at 13,000 rpm for 10 min. The supernatant was applied to His·Bind resin (Novagen, Madison, WI) and bound proteins were eluted with 1 × elution buffer (1 M imidazole, 0.5 M NaCl, 20 mM Tris-HCl, pH 8.0). The purity of the eluted protein was confirmed by 12% or 15% SDS-PAGE.

### DNaseI Footprinting Assay

To determine the H-NS binding site on the upstream region of *leuO*, the end-labeled 308-bp DNA fragment of the *leuO* promoter region (nucleotides -45 to -352 relative to the translation initiation site) was amplified using primers FP-leuO-F and ^32^P-labeled FP-leuO-R. To investigate the binding competition of ToxR, LeuO and H-NS on the upstream region of *leuO*, an end-labled 173-bp DNA fragment of the *leuO* promoter region (nucleotides -180 to -352 relative to the translation initiation site) and an end-labled 279-bp DNA fragment of the *leuO* promoter region (nucleotides -45 to -323 relative to the translation initiation site), were amplified using primers FP-leuO-F2 and ^32^P-labeled FP-leuO-R2, FP-leuO-F3 and ^32^P-labeled FP-leuO-R, respectively. Two hundred ng of the amplified *leuO* promoter region was incubated with increasing amounts of purified ToxR, LeuO, and H-NS at 30°C for 30 min in 50 μl of buffer (10 mM HEPES, 100 mM KCl, 200 μM EDTA, 10% glycerol, pH 7.5). After 30 min, 50 μl of CaCl_2_-MgCl_2_ solution (10 mM MgCl_2_, 5 mM CaCl_2_) was added to the reaction. Then, 0.25 U of DNase I (Promega, Madison, WI) was added and the reaction was incubated at room temperature for 1 min. These reactions were terminated by the addition of 90 μl stop solution (200 mM NaCl, 30 mM EDTA, 1% SDS). After the addition of 500 μl ethanol, samples were precipitated on ice for more than 30 min and centrifuged. DNA pellets were washed with 70% ethanol, and resuspended in 10 μl loading buffer [0.1 M NaOH:formamide (1:2), 0.1%xylene cyanol, 0.1% bromophenol blue]. The samples and the sequencing ladder generated with ^32^P-labeled FP-leuO-R and ^32^P-labeled FP-leuO-R2 primers were denatured for 5 min at 95°C, chilled on ice, and loaded onto a 6%sequencing gel. The sequencing ladders were prepared using an AccuPower DNA sequencing kit (Bioneer, Daejeon, Korea).

## Results

### H-NS Represses the Expression of *leuO*

**Fig. 1:**
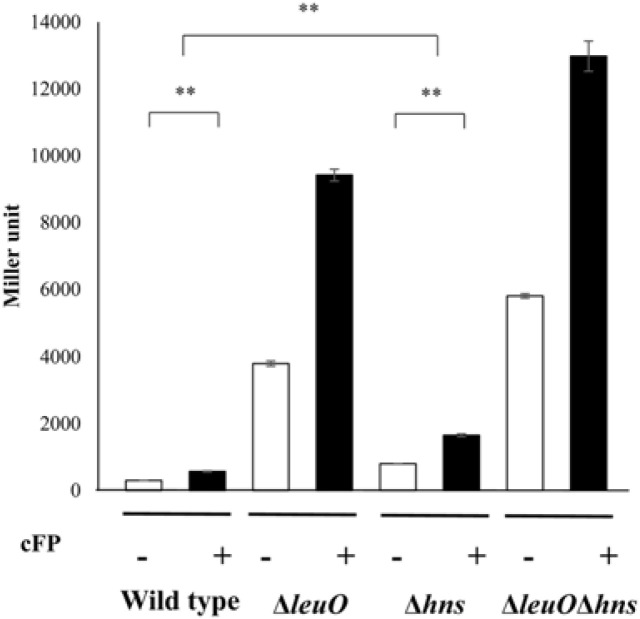
H-NS represses the expression of *leuO*. β-Galactosidase activity of *leuO*-*lacZ* fusion in the absence (open bars) and presence (solid bars) of exogenous 5 mM cFP in wild type *V. vulnificus*, *leuO*-deletion isotype *hns*-deletion isotype, and *leuO*hns-double deletion isotype. Error bars denote standard deviations of the results of three independent experiments (**p < 0.005 in Student’s t test).

**Fig. 2:**
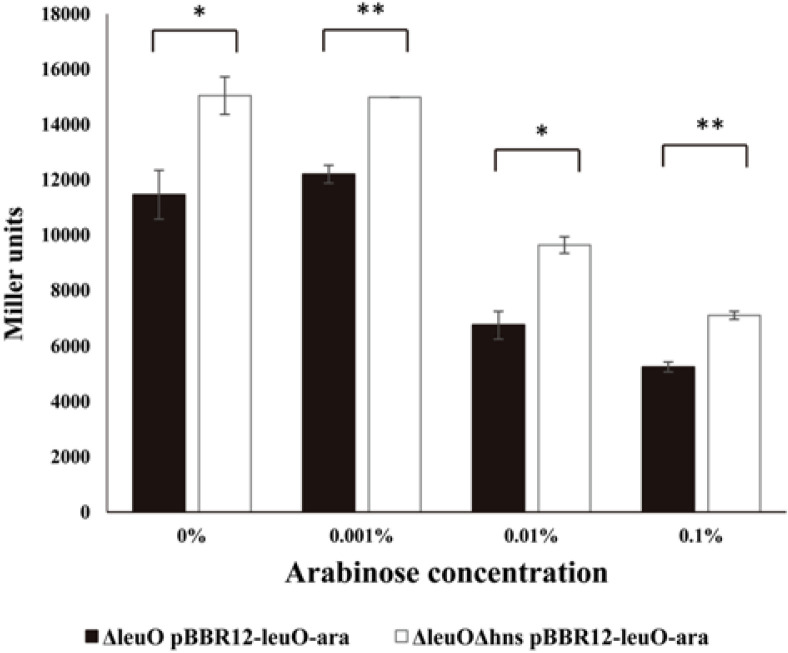
Transcription levels of *leuO* regulated by various concentrations of LeuO in the presence or absence of the H-NS. Transcription levels of *leuO* as measured by β-galactosidase activities from Δ*leuO*(pBBR12-leuO-ara) (solid bars) or Δ*leuO*Δ*hns*(pBBR12-leuO-ara) (open bars) harboring pMZtc-*leuO*. Culture conditions are described in Materials and Methods. To induce the expression of LeuO, the culture was split into four aliquots after reaching an A_600_ of 0.4, and then various concentration of L-arabinose were added (0, 0.001, 0.01, 0.1%). Error bars denote standard deviations of three independent experiments (**p < 0.005; *p < 0.05 in Student’s *t* test).

Our finding that the upstream region of *leuO* has a high A+T content led us to assume that H-NS may be involved in the regulation of the expression of the master regulator. Our previous transcriptional fusion assay of *leuO* revealed that the transcription level of *leuO* increased in the presence of cFP [7]. We thus quantitatively measured the expression level of *leuO* using a *lacZ*-transcriptional fusion construct in a wild type strain, an *hns*-null mutant, the *leuO*-null mutant, and an *leuO*/*hns* double mutant, in the presence of, or absence of, exogenous cFP. β-Galactosidase levels were higher with the addition of cFP in all tested strains. The expression level was enhanced in the *hns*-null mutant and even higher in the *leuO*/*hns* double mutant when compared to the wild type strain irrespective of cFP (Fig. 1). Our previous study demonstrated that β-galactosidase activity from a chromosomal *leuO*-*lacZ* fusion, in a Δ*leuO* isotype harboring an L-arabinose-inducible LeuO overexpression vector, gradually decreased when the concentration of L-arabinose was increased [7]. We then measured the β-galactosidase activity of *leuO*-*lacZ* fusion under the same conditions in the presence and absence of H-NS. The same effect of an L-arabinose-inducible LeuO was observed in the Δ*leuO*Δ*hns* isotype, but the overall quantity of *leuO* transcriptions was higher in Δ*leuO*Δ*hns* than in Δ*leuO* (Fig. 2). These results indicate that H-NS represses the expression of *leuO* in *V. vulnificus*.

### H-NS Binds to the Upstream of *leuO*

**Fig. 3:**
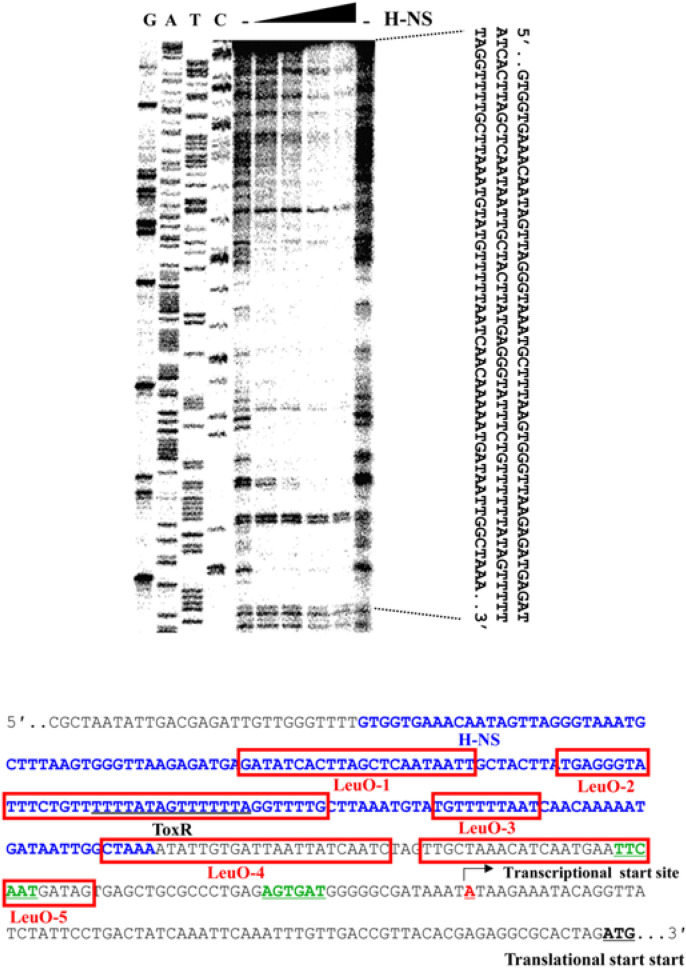
The region upstream of *leuO* is bound by H-NS. DNaseI protection assay of the region upstream to *leuO* using purified H-NS. 200 ng of labeled *leuO* promoter DNA was combined with either no H-NS, lanes 1 and 6; or 0.5 μM, 1 μM, 2 μM, and 5 μM H-NS, lanes 2-5, respectively. The nucleotide sequences protected by H-NS indicated as blue letters and those protected by LeuO boxed in red color are indicated on the genetic map.

H-NS is known to function as a nucleoid organizer and a transcriptional silencer at promoters exhibiting AT-rich highly curved DNA [3]. We predicted that H-NS represses the expression of *leuO* by binding to a *cis*-element in the upstream region of *leuO*. To confirm this hypothesis, we performed DNaseI footprinting analysis. As shown in Fig. 3, H-NS binds to a long stretched region (-251 ~ - 90 with respect to the translation start site) in the upstream of *leuO*, which includes the binding site for ToxR as well as those for LeuO; LeuO-1 through 4 [7](Fig. 3).

### H-NS Inhibits the Binding of ToxR to the *cis*-acting Element of *leuO*

**Fig. 4:**
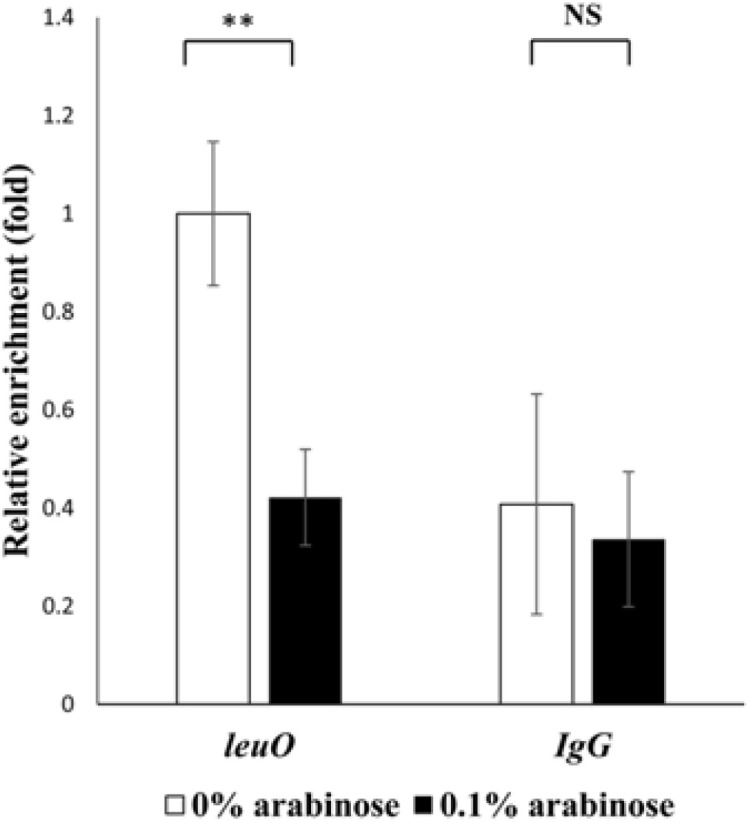
ChIP analysis showing that H-NS inhibits ToxR binding to *cis*-acting elements upstream of *leuO*. The *toxRS*/*hns* null mutant cells expressing strep-tagged ToxR were cross-linked, washed, and sonicated as described in Materials and Methods. Lysates were then treated with either anti-Strep-tag II monoclonal antibody or normal mouse IgG as a control. Free DNA was purified and analyzed by quantitative real-time PCR (qRT-PCR) on a Light Cycler 480 II real-time PCR system. Relative enrichment was calculated as the amount of transcript compared to the transcript from cells without L-arabinose. Values are averages from biological experiments done in triplicate. Error bars indicate standard deviations (**p < 0.005; NS, not significant in Student’s *t* test with *p* > 0.05).

ToxR is essential for the expression of LeuO in *Vibrio* spp [5, 6, 7]. We had previously defined the ToxR binding site of the upstream region of *leuO* by DNaseI footprinting analysis [7]. As shown Fig. 3, the ToxR binding site overlaps with the H-NS binding site on the upstream region of *leuO*. This result suggests that H-NS can inhibit the binding of ToxR to the ToxR box upstream to *leuO*. To confirm this hypothesis, we performed ChIP analysis using an antibody against ToxR. As shown in Fig. 4, we observed a lower level of binding of ToxR to the *leuO* upstream region in the presence of H-NS than in the absence of H-NS. Under the same conditions, no significant difference was observed in the negative control sample that treated with a non-specific IgG. These results suggest that high amounts of the H-NS inhibits the ToxR binding to the *cis*-acting element of *leuO*.

### ToxR Outcompetes LeuO and H-NS at the Same Molarity

**Fig. 5:**
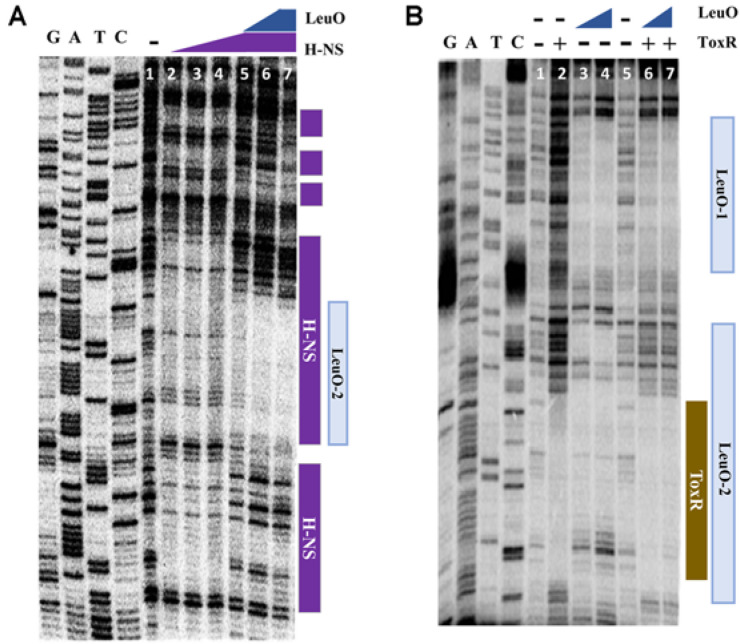
Comparison of the binding of LeuO, H-NS, and ToxR at various concentrations to the upstream region of *leuO*. (**A**) DNaseI protection assay of the region upstream of *leuO* using purified LeuO and H-NS; Lane 1, no protein was added; Lanes 2-4, 0.5, 1, and 2 μM of H-NS, respectively, without LeuO; Lanes 5-7, 0.5, 1, and 2 μM of LeuO, respectively, with 2 μM of H-NS. In all lanes, 200 ng of the DNA fragment with 3’- labeled *leuO* promoter region was added. (**B**) DNaseI protection assay of the region upstream of *leuO* using purified LeuO and ToxR; Lanes 1 and 5, no protein was added; Lanes 2, 3 μM of ToxR; Lanes 3-4, 2 μM and 5 μM of LeuO, respectively, without ToxR; Lanes 6-7, 2 μM and 5 μM of LeuO, respectively, with 3 μM of ToxR were incubated. In all lanes, the DNA fragment with 200 ng of 3’- labeled *leuO* promoter region was added.

**Fig. 6:**
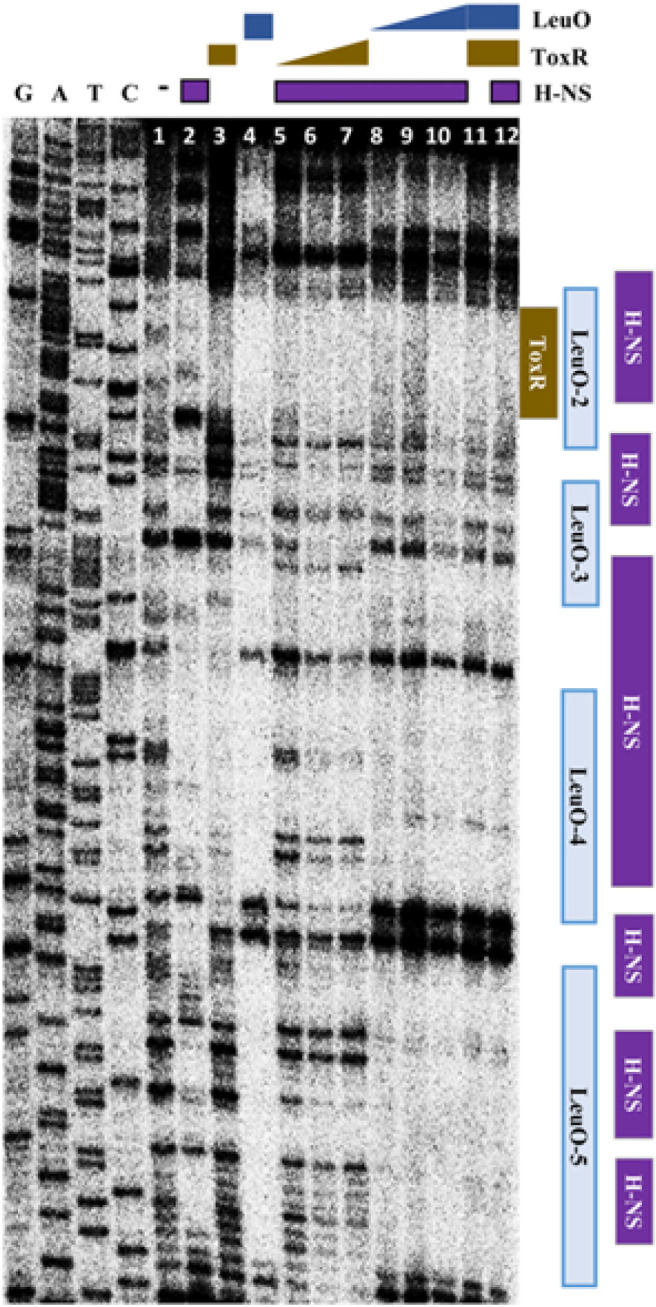
Binding of LeuO, H-NS, and ToxR to the region upstream of *leuO*. DNaseI protection assay of the region upstream of *leuO* using purified H-NS, LeuO, and ToxR at various concentrations; Lane 1, no protein was added; Lanes 2-4, 1 μM of H-NS, ToxR, and LeuO, respectively; Lanes 5-7, 0.2 μM, 0.5 μM, and 1 μM of ToxR, respectively, with 1 μM of H-NS; Lanes 8-10, 0.2 μM, 0. 5 μM, 1 μM of LeuO, respectively, with 1 μM of H-NS; Lane 11, 1 μM of ToxR and LeuO; Lane 12, 1 μM of LeuO, ToxR and H-NS. In all lanes, the DNA fragment with 200 ng of 3’- labeled *leuO* promoter region was added.

As illustrated in Fig. 3, H-NS binds to wide regions which overlap with binding sites for LeuO as well as for ToxR. We assessed the binding of ToxR, LeuO, and H-NS at various concentrations to the upstream region of *leuO* using footprinting analysis. As shown in Fig. 5A, binding of LeuO interferes with the binding of H-NS at the same molarity, suggesting that the binding affinity of LeuO in the region is stronger than that of H-NS. We also compared the binding of LeuO and ToxR. As shown in Figs. 5B and 6, ToxR outcompetes with LeuO for binding on overlapping binding regions, suggesting that the binding affinity of ToxR is stronger than LeuO. Furthermore, when ToxR binds to the upstream region of *leuO*, the pattern of H-NS binding changes somewhat (Fig. 6). These results indicate a binding affinity for the upstream region of *leuO* in the order of ToxR, LeuO, and H-NS at the same molarity.

### Cis-acting Elements Upstream to the *leuO* Genes Are Highly Conserved among *Vibrio*naceae

**Fig. 7:**
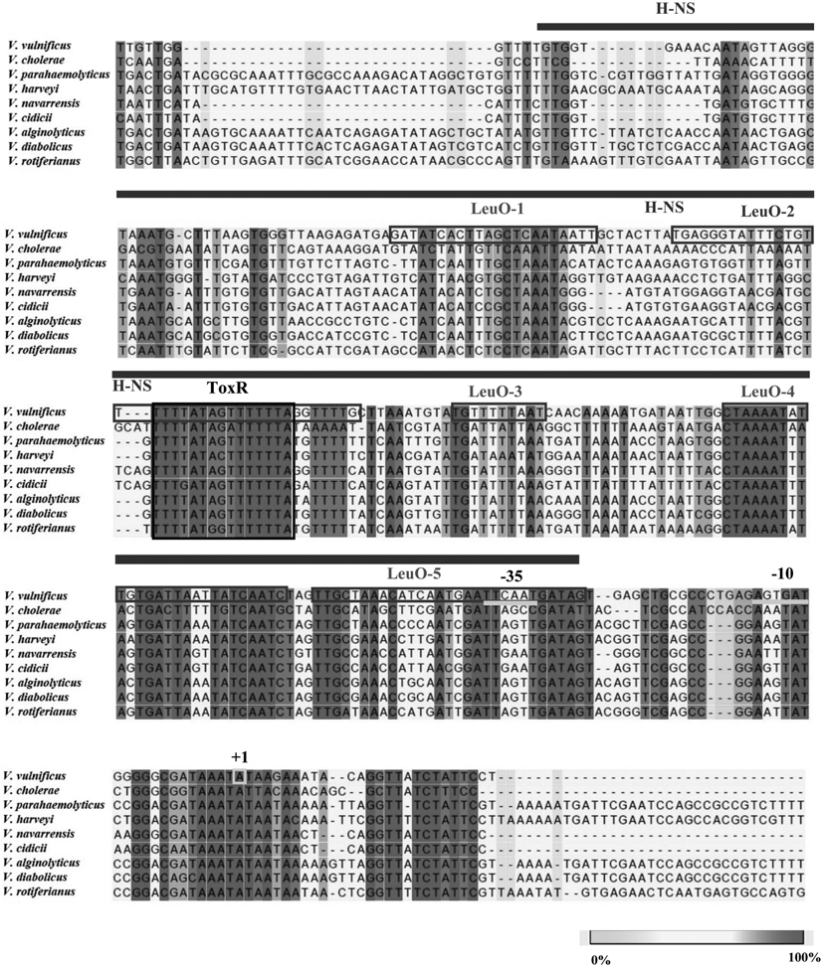
DNA sequence alignment of regions upstream of *leuO* genes in *Vibrio* spp. Nucleotide sequences were aligned using a CLC sequence viewer. The transcription start site (+1) and promoter regions (-35 and -10) are indicated. Binding sites for ToxR, LeuO, and H-NS are also indicated. Strains from which these *leuO* sequences originated and the associated NCBI accession numbers are as follows: *V. vulnificus* MO6-24/O (VVM06_02645), *V. cholerae* El Tor N16961 (VC2485), *V. parahaemolyticus* RIMD 2210633 (VP0350), *V. harveyi* ATCC 43516 (AL538_RS12030), *V. navarrensis*ATCC 51183 (EA26_RS02630), *V. cidicii* 2756-81 (AUQ44_RS10800), *V. alginolyticus* K10K4 (K10K4_RS01815), *V. diabolicus* FDAARGOS_105 (AL537_06395), *V. rotiferianus* B64D1 (BSZ04_RS21800).

DNA nucleotide sequences in regions upstream to the *leuO* genes were compared among species belonging to *Vibrio*naceae. As shown in Fig. 7, these regions show a high similarity. Especially, the ToxR binding sequences show a high identity. LeuO binding sites also are well conserved, and sequences for H-NS also are conserved, suggesting that regulatory modes of *leuO* genes among these species are highly homologous.

## Discussion

Recent findings have demonstrated that LeuO is a master regulator of the cFP-mediated signaling pathway, which plays an important role in the pathogenesis of pathogenic vibrios. [7, 8]. ToxR directly regulates its own target genes, such as virulence factors *ompU* [5] and *ctxAB* [6, 19]. ToxR also transduces the cFP-triggering signal to LeuO [5, 6]. LeuO not only directly regulates its targets, including LeuO itself, and a series of other genes [20], but also relays the cFP-signal to downstream regulators vHU*αβ*, and consequently to the alternative sigma factor RpoS, each of which also regulates its own target genes. Those genes are of importance in survival and propagation in host environments. Therefore, the expression level of LeuO is crucial for the pathogen, and must be coordinated by fine-tuning through multiple factors, including *cis*- and *trans*-elements, dependent upon numerous environmental factors [7]. Such *trans*-acting elements include cFP-dependent acting ToxR, and LeuO itself [8], as well as iron and fur (unpublished results). This study has added H-NS as another factor to the previous list.

H-NS is known to bind to AT-rich DNA sequences [21, 22], rendering the target genes transcriptionally inactive or silent [1, 23]. Because there are many AT-rich sequences on the upstream sequence of *leuO*, we had expected that H-NS would polymerize along the *leuO* upstream region, and this study has proved our hypothesis. H-NS in excessive quantities antagonizes the action of ToxR, but the action is outcompeted by ToxR at the same molarity, suggesting that H-NS may function only when cFP or other signals which activate ToxR, are not present. It is likely that, under conditions where the expression of *leuO* is not required (or even harmful to cells), H-NS plays a role as a basal stopper. It also is possible that H-NS may assist with a loop-formation, which would facilitate the immediate expression of *leuO* when necessary.

**Fig. 8:**
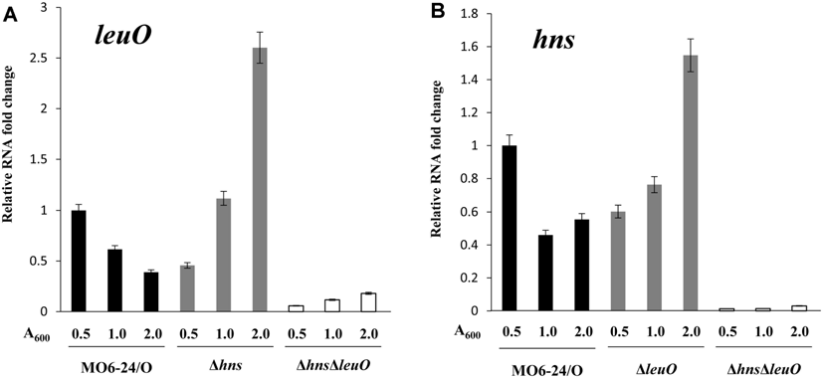
Expressions of *leuO* and *hns* are antagonized by each other. Comparisons of transcriptional level of *leuO* (**A**) and *hns* (**B**) in wild-type *V. vulnificus* MO6-24/O, Δ*leuO*, Δ*hns*, Δ*leuO*Δ*hns* at early exponential phase (A_600_ = 0.5), late exponential phase (A_600_ = 1.0), and stationary phase (A_600_ = 2.0). Overnight culture cells were sub-cultured into LB medium and grown to each phase. RNA levels were quantified by qRT-PCR using primers shown in Table 2S. Comparative threshold cycle (ΔCt) method was employed for quantification, and RNA-fold change was normalized to the value for MO6-24/O. The data are average values from three independent samples, and error bars denote the standard deviations.

The expression of H-NS is also repressed by LeuO (Fig. 8) [24], suggesting that H-NS and LeuO antagonize each other to form feedback inhibition. Such a regulatory circuit appears to attributes to the fine coordination of LeuO expression, and such a pattern may prevent drastic changes in intracellular LeuO levels.

In summary, H-NS regulates the expression of *leuO* as a basal stopper by direct binding, whereas the binding affinity of H-NS to the upstream region of *leuO* is lower than those of ToxR and LeuO in *V. vulnificus*; with this pattern of regulation being likely a common feature of *leuO* expression in *Vibrio*naceae.

## Supplemental Materials

Supplementary data for this paper are available on-line only at http://jmb.or.kr.
