# Supplementary material for: H-NS Silences Gene Expression of LeuO, the Master Regulator of the Cyclic(Phe-Pro)-dependent Signal Pathway, in Vibrio vulnificus
Source: J Microbiol Biotechnol. 2020 Mar 27;30(6):830–8. doi: 10.4014/jmb.2001.01021 (PMC9728156; doi:10.4014/jmb.2001.01021)
Supplement: Supplementary file 1 [file JMB-30-6-830-supple.pdf]

J. Microbiol. Biotechnol.
https://doi.org/10.4014/jmb.2001.01021

Table S1. Bacterial strains and plasmids used in this study.

| [illegible]ns or plasmids | Genotypes | Sources |
|---|---|---|
| **Strains** | | |
| ***E. coli*** | | |
| DH5α | $\lambda^-$φ80d*lacZ*Δ*M15*Δ(*lacZYA-argF*)*U169 recA1 endA1 hsdR17* ($r_K^- m_K^-$)*supE44 thi-1 gyrA relA1* | Our collection |
| S17-1 | [C600::RP4-2 (Tc::Mu)(Km::Tn7) *thi pro hsdRM*$^+$ *recA* | 1 |
| S17-1 λ *pir* | S17-1 with λ*pir* lysogen | 1 |
| BL21(DE3) | $F^-$*ompT hsdSB* ($r_B^-$,$m_B^-$)*gal dcm* (DE3) | Novagen |
| GI698 | $F^-$ $\lambda^-$ *lacIqlacPL*8 *ampC*::Ptrp cI | 2 |
| ***V. vulnificus*** | | |
| MO6-24/O | Pathogenic clinical isolate | 3 |
| Δ*leuO* | Derivative of MO6-24/O with a deletion in *leuO* | This study |
| Δ*hns* | Derivative of MO6-24/O with a deletion in *hns* | This study |
| Δ*leuO*Δ*hns* | Derivative of MO6-24/O with double deletions in *leuO* and *hns* | This study |
| Δ*toxRS*Δ*hns* | Derivative of MO6-24/O with triple deletions in *toxRS* and *hns* | This study |
| **Plasmids** | | |

| | | |
|---|---|---|
| pGEM-T easy | TA cloning vector, *lacZ*, f1 origin, Ap$^r$ | Promega |
| pGEM-hns-up | pGEM-T easy vector containing *leuO* upstream region | This study |
| pGEM-hns-down | pGEM-T easy vector containing *leuO* downstream region | This study |
| pDM4 | Suicide vector for an allelic exchange, *sacB*, Cm$^r$ | 4 |
| pDM4-d*hns* | pDM4 with a deletion in *hns* of *V. vulnificus* | This study |
| pDM4-d*leuO* | pDM4 with a deletion in *leuO* of *V. vulnificus* | This study |
| pMZtc-*leuO* | pMZtc with a promoter region of *leuO* | 5 |
| pET21a | Expression vector, C-terminal His-tag, Ap$^r$ | Novagen |
| pET-HNS | pET21a vector with *V. vulnificus* H-NS | This study |
| pET-ToxR-N | pET21a vector with *V. vulnificus* N-terminal domain of ToxR | 5 |
| pRE1-*leuO* | *leuO* expression vector | 6 |
| pBBR12-*leuO*-ara | pBBR-MCS2 vector with the *V. vulnificus leuO* under the *araC* promoter | 5 |
| pBAD-TOPO | Arabinose-regulated expression plasmid, Ap$^r$ | Invitrogen |
| pBBR12-hns-ara | pBBR-MCS2 vector with the *V. vulnificus hns* under the *araC* promoter | This study |


1. Simon R, Priefer U, Pühler AA. 1983. broad host range mobilization system for *in*

*vivo* genetic engineering: transposon mutagenesis in gram negative bacteria. *Nature Biotechnol.* **1:** 784-791

2 Lavallie ER, DiBlasio EA, Kovacic S, Grant KL, Schendel PF, McCoy JM. 1993. A thioredoxin gene fusion expression system that circumvents inclusion body formation in the *E. coli* cytoplasm. *Nature Biotechnol.* **11**: 187-193

3 Reddy GP, Hayat U, Abeygunawardana C, Fox C, Wright AC, Maneval DR, *et al.* 1992. Purification and determination of the structure of capsular polysaccharide of *Vibrio vulnificus* M06-24. *J. Bacteriol.* **174**: 2620-2630

4 Milton DL, O'Toole R, Horstedt P, Wolf-Watz H. 1996. Flagellin A is essential for the virulence of *Vibrio anguillarum*. *J. Bacteriol.* **178**: 1310-1319

5. Park NY, Kim H, Wen Y, Lee KW, Lee S, Kim JA, *et al*. 2019. Multi-Factor Regulation of the Master Modulator LeuO for the Cyclic-(Phe-Pro) Signaling Pathway in *Vibrio vulnificus*. *Sci Rep.* 9: 20135

6. Kim JA, Park JH, Lee MA, Lee HJ, Park SJ, Kim KS, *et al.* 2015. Stationary-phase induction of *vvpS* expression by three transcription factors: repression by LeuO and activation by SmcR and CRP. *Mol. Microbiol.* **97:** 330-346

Table S2. Primers used in this study.

| Function and Name | Nucleotide sequence (5′ → 3′) |
|---|---|
| **Construction of a *leuO*-deletion mutation** | |
| dleuO-up-F | AATCCCGGGAGAGCTCTTGGCTTAGCGACGGTGA |
| dleuO-up-R | CAACCACTTCGCCACAGCTCATCGCATCTTTTTATC |
| dleuO-down-F | AAAAGATGCGATGAGCTGTGGCGAAGTGGTTGCTGCG |
| dleuO-down-R | CGGGTAACCTGAGCTCATCGAAGATTTTCACTGC |
| **Construction of a *hns*-deletion mutation, a *leuO/hns* deletion mutation, and *toxRS/leuO* double mutations** | |
| dhns-up-F | CCTGAGCATGCGGATGTCCGTTACTTATG |
| dhns-up-R | TCAGGATCCCAACAGAGTTTTTGTTAG |
| dhns-down-F | GTCGGATCCGCGGGTAAATCTCTAGAAGAT |
| dhns-down-R | TACCTCGAGGTTGTTTTGACGCTTGGC |
| **Construction of an arabinose-inducible H-NS expression system** | |
| BAD-hns-F | ATGTCTGAACTAACAAAAACTC |
| BAD-hns-R | TTAGATTTCGAAATCTTC |
| pBAD-F | ATGCCATAGCATTTTTATCC |
| pBAD-R | GATTTAATCTGTATCAGG |
| **qRT-PCR for quantitative analysis of *leuO* and *hns* transcripts** | |
| leuO_qPCR_F | TCAAACTGGCGATTTGCAGC |
| leuO_qPCR_R | AGGATGCTGCTTAGACGTCAC |
| HNS_qPCR_F | GGAAGCAGAAGAACGTGCAG |

| | |
|---|---|
| HNS_qPCR_R | AGCTTGGATTGCAGAAGGTGT |
| **ChIP analysis** | |
| leuO-chIP-F | CGGAGTGGATCTCAACCTACTGAC |
| leuO-chIP-R | ACGCATGAAAAGCTCATCATTAAA |
| **Expression of a recombinant H-NS** | |
| Pet-hns-F | GGATCCATGTCTGAACTAACAAAAAC |
| Pet-hns-R | GTCGACGATTTCGAAATCTTCTAG |
| **DNaseI footprinting** | |
| FP-leuO-F | CGCTAATATTGACGAGATTG |
| FP-leuO-F2 | GGTACCCGCTAATATTGACGA |
| FP-leuO-F3 | GTGGTGAAACAATAGTTAG |
| FP-leuO-R | GTCAGGAATAGATAACCTG |
| FP-leuO-R2 | TTTGTTGATTAAAAACAT |

Nucleotides modified for the generation of restriction sites are underlined.
